# Development of Tools to Understand the Relationship between Good Management Practices and Nest Losses in Meliponiculture: A Pilot Study in Latin American Countries

**DOI:** 10.3390/insects15090715

**Published:** 2024-09-19

**Authors:** Joseline Sofía Ocaña-Cabrera, Sarah Martin-Solano, Claude Saegerman

**Affiliations:** 1 Research Unit of Epidemiology and Risk Analysis Applied to Veterinary Sciences (UREAR-ULiège), Fundamental and Applied Research for Animal and Health (FARAH) Center, Department of Infections and Parasitic Diseases, Faculty of Veterinary Medicine, University of Liege, 4000 Liège, Belgium; jocana@doct.uliege.be; 2 Grupo de Investigación en Sanidad Animal y Humana (GISAH), Carrera de Ingeniería en Biotecnología, Departamento de Ciencias de la Vida y de la Agricultura, Universidad de las Fuerzas Armadas ESPE, P.O. Box 171-5-231, Sangolquí 171103, Ecuador; ssmartin@espe.edu.ec

**Keywords:** stingless bees, management, practices, biosecurity, nest loss, Latin America, evaluation tools

## Abstract

**Simple Summary:**

The overall decline of bees may be exacerbated by the simultaneous presence and interaction of multiple causal factors. To elucidate how these factors interact and their collective impact, it is of the utmost importance to develop effective analytical tools. We collected data through an online questionnaire. We started estimating the annual mortality of stingless bee nests at 15%. Four risks to stingless bee survival were identified: invasive species (73%), the proximity of nests to sources of environmental pollution (61%), the presence of honey bees as potential transmitters of diseases (57%), and unusual behavior reports (44%). The biosecurity practices with the highest compliance rates were hand washing (79%), sterilization (75%), storage conditions for product quality (66%), and the use of protective equipment (40%). The spider web and barometer tools facilitate a unified observation of the status of implementation or non-implementation of biosecurity measures, actions to care for the environment in which stingless bees live, the quality and efficiency of nest management techniques, and the monitoring of the health status of stingless bees. The comprehensive evaluation of these factors within best management practices (BMPs) facilitates immediate decision-making and the implementation of enhancements, as well as individual and collective feedback.

**Abstract:**

Insect pollination services amount to USD 235–577 billion. Seventy five percent of agricultural production for human consumption depends on pollination, mainly by bees. A decline in pollinators, including Meliponini tribe bees, will impact the economy, food security, human health, and ecosystem stability, especially in tropical forests where stingless bees are the main pollinators. The objective of this survey was to understand the relationship between good management practices and nest losses in meliponiculture, encompassing biosecurity and conservation criteria. A 36-question survey was organized and spread. We received 92 responses, representing 4548 managed nests. The primary motivation for engaging in meliponiculture was biodiversity conservation (92%). More than 50% of the questions on biosecurity were answered as “applied”. Hand washing before any activity with bees was the main rule, followed by material sterilization and personal protective equipment use. The annual mortality rate of stingless bee nests was estimated at 15%. Nest invaders (72%) and nearby sources of pollution (60%) were identified as the main potential causes of nest losses. From a general perspective, meliponiculture practices continue to expand remarkably. The implementation of effective nest management strategies is associated with a reduction in nest losses. It is important to consider One Health’s perspective to ensure optimal management practices.

## 1. Introduction

The global economic value of pollination services amounted to USD 235–577 billion, representing 10% of the total value of agricultural production for human consumption in 2021. Around 75% [1] of this agricultural production depends on pollinators, especially bees [2]. It is evident that the decline of main pollinators, including stingless bee species [3], will have a great economic impact on food security, human health, and ecosystem stability.

The available data indicate that the Neotropics are home to more than 15,150 species of bees [4], and it is only a third of the total animal species richness in that region. Worldwide, the number of stingless bee species exceeds 500 [5]. There is the possibility of finding subspecies or cryptic species due to the complexity of certain genera such as *Melipona beecheii* [6] or the taxonomic updating of stingless bees [7]. In Ecuador, a great contribution showed the presence of >200 [8,9], consolidating the megadiverse label despite the small size of the country with other neighbors.

There are multiple approaches to practicing meliponiculture, and they are contingent upon the motivations, needs, and objectives of the practitioner [10]. Meliponiculture represents a fusion of ecological (from the academy) and cultural (empirical local) knowledge, and both, along with stingless bees, serve as interesting fusion that facilitates the transition to sustainable practices within complex farming systems [11].

The five major threats for native tropical bees are deforestation, agriculture intensification, the spread of exotic species [12], climate change, and resource–habitat loss [13]. The introduction of non-native pollinators modifies socioecological interactions between insects and environmental health, i.e., by competing with native insects for floral resources or due to the spread of new diseases [14] for which the native insects have no immune defense [15]. The effects of deforestation include habitat loss and fragmentation [16], which are mainly caused by the expansion of crops such as potatoes in the Colombian and Ecuadorian Andes [17], soybeans in the Brazilian Amazon rainforest [18], or the expansion of areas focused on cattle breeding [12]. 

Meliponiculture practices that include harvesting honey and pollen, dividing nests, and selling nest products have faced several other menaces, such as the loss of numerous daughter colonies from a single mother, inbreeding, and queen succession problems in *Scaptotrigona* and *Cephalotrigona* species [19]. There are mainly two stingless bee nest invasive insect problems. The first, *Lestrimelitta* sp., is a kleptobiotic stingless bee, considered a resource thief that uses a chemical trickery mechanism based on its cuticular characteristics [20]. The other major invasive insect problem is Phoridae flies (*Pseudohypocera kerteszi*), which, avoiding the guardians at the nest entrance, lay eggs in pollen pots, containers, and near the brood, which will develop into white larvae that feed on the bee bread [21]. 

A study of the population dynamics of stingless bees in seasonal dry lowlands in Costa Rica reveals that they invest more efforts in colony survival rather than in increasing their reproductive rates, which means that, under better life conditions, these stingless bees can survive around 23 years [22], but the most recent study of colony loss in Latin America indicated a 39.6% loss of stingless bee colonies per year across the region. Furthermore, the study found that losses were highest in summer and increased with farm size [23]. These findings suggest that maintaining the overall health of bee colonies is challenging, which could have significant implications for the economic survival of stingless bee keepers. The role of stingless bee keepers is an option to care for intangible heritage and the conservation of natural resources [9], as well as their training and adoption of best practices to preserve the life of stingless bees and thus the environment. 

The FAO, the WHO, and the European Commission have recognized good farming practices in beekeeping and describe their advantages, such as improved bee colony health, decreased medicinal costs, increased hive production, and the yield of healthier and higher quality honey [24]. In this sense, stingless beekeeping also needs the application of good management practices, since it has been recognized as an informal activity with poor management [25] which continues to grow and expand, especially in Latin America, at an accelerated rate [26,27,28,29]. Good practices in the management of stingless bees are a means to reduce risks associated with human error that impact human public health due to the consumption of nest products, such as honey, contaminated with agrochemicals [30]. In addition, the same risks can affect bee health, as pesticide residues can bioaccumulate in bees’ bodies, in their food, and in nest structures, affecting their health, condition, and ability to survive.

Ecuadorian meliponiculture has developed depending on the climatic region. The southern highlands region, especially the province of Loja, has the highest development at the national level in dry tropical forest meliponiculture, followed by Amazon rainforest meliponiculture, urban tourist-productive meliponiculture in the coastal region, and conservations projects in protected areas.

In terms of regulations on stingless bee products, the Ecuadorian Service of Normalization (INEN) does not contemplate quality standards for pot honey or pot pollen [31]. Regarding good management practices, the Agency for Regulation and Phytosanitary—Zoo Sanitary Control (AGROCALIDAD) has only issued beekeeping guidelines [32]. In terms of bee health, the capital of the country, Quito, recently issued an ordinance banning some herbicides and pesticides [33].

A more comprehensive approach to the assessment of the impact of stingless bee breeding and management is required, encompassing social, ecological, and cultural dimensions. This approach will facilitate the development of more effective pollinator-friendly strategies and diversified agricultural systems [34].

Thus, in response to the need to develop tools to improve decision-making and provide guidance for practical actions to reduce and prevent pollinator decline, this survey aims to (i) collect stingless bee keepers’ knowledge about the management of stingless bee nests (from the origin of the nest to the harvesting of products); (ii) estimate the nest death rate; (iii) identify specific health risk factors for stingless bee nests; and (iv) develop tools to correlate the application of good management practices with nest losses.

## 2. Materials and Methods

### 2.1. Online Survey Development

The free software KoboToolbox (v2022 1.2.) was used to prepare an online questionnaire with 36 questions (Table S1). All questions were configured as mandatory to ensure that all were answered. The anonymity of respondents was maintained. The survey was organized into 4 sections: (i) socio-demographic variables, (ii) biosecurity and product management, (iii) nest management and infrastructure of the farm, and (iv) sanitary and environmental aspects. The questions used for nest death rate estimation were not included in any of the previous groups since the data obtained were directly processed with the formula in Section 2.3 (namely, “Statistical Analysis”). The types of questions included in the questionnaire were single-choice, multiple-choice, and open-ended questions. The survey was available from 23 March 2022 to 31 December 2022, in two languages: Spanish and Portuguese. The target audience was meliponicultors (stingless bee keepers) with experience in managing at least one (1) nest of any stingless bee species in any country of Latin America.

Before the public launch, the questionnaire was reviewed by three experienced stingless bee keepers. They gave points for improvement and suggestions for the survey, for a better understanding of the target audience. After adding these modifications, the survey was officially launched online. The survey link (https://ee.kobotoolbox.org/x/HVbthWiD, accessed on 31 July 2023) was disseminated through social networks (meliponicultors’ groups on WhatsApp and Facebook) as well as through e-mails sent to local meliponiculture organizations (when available) and to the authors of scientific articles related to stingless bees. The rationale behind selecting this particular methodology for the survey spread is twofold. Firstly, this is a pilot study designed to test the operationality of a data relation–visualization tool. Secondly, according to the Ecuadorian Observatory of Information and Communication Technologies (TIC), 82.88% of citizens in rural areas with access to a phone use social networks as their primary source of information. Together with Brazil, Colombia, Costa Rica, and México are included in the medium- and high-Significant Rural Connectivity Index countries [35]. Third, without a national official registry of meliponicultors, we used social networks as a census tool.

### 2.2. Scoring System Development

The questions in section (i), socio-demographic variables, and other open-ended questions of the inquiry were not included in the subsequent phase of the study.

All answer options, from single-choice and multiple-choice questions, were numerically scored by the authors. The lowest score represented the “worst situation” and the highest score represented the “best situation”. The criteria for this scoring considered those answers that were based on scientific evidence and focused on the conservation and guarantee of the best living conditions for stingless bees as a priority and of greater weight. In addition, a consensus was reached among a panel of four experts in biology, epidemiology, meliponiculture, and biosecurity. The panel agreed on the options for each question, from “worst situation” to “best situation”. 

Each question had different maximum scores. Each section—(ii) biosecurity and product management, (iii) nest management and infrastructure of the farm, and (iv) sanitary and environmental aspects—had a different number of questions. To ensure the fairness, consistency, and accuracy of the weighting of each section on the results, the maximum score was normalized and the minimum difference in the number of questions within each section was targeted.

### 2.3. Statistical Analysis

Questions were classified into five groups, one including socio-demographic information (INF) and four explaining the application of good management practices (GMP) in meliponiculture: (i) environment and conservation (ENV PROTEC), (ii) producer training and modern techniques (TECHN), (iii) the use of personal protective equipment and biosecurity measures (BIOSEC), and (iv) health care (HEALTH).

The scoring of the questions was applied to those from which quantitative information could be obtained. The maximum was calculated for each question based on the response options and we categorized these options as “best” if they adhered to conservation criteria and “worst” if they were far from it (called “theoretical best score”). To verify the analyses, the same procedure was performed, except that the maximum this time was taken according to the “best” answer given by the respondents (called “best meliponicultor score”).

An overall score for each respondent was calculated using the sum of scores obtained for all their responses and the sum of the “best” scores for each question (for explanation, see Table S1).

The calculation of the nest death rate (NDR) of stingless bees was calculated as follows according to the formula modified from [36]:

(1) $$Nest\;death\;rate\;(NDR)=\frac{\#nest\;dead}{\#nest\;until\;2021+\#nest\;IN+\#nest\;OUT}$$

The terms inside the numerator and denominator are explained as follows:

#nests dead—the number of nests of stingless bees that died the last year (question (Q) 28);

#nests until 2021—the number of nests of stingless bees that existed until 2021 (Q 27);

#nests IN—the number of nests of stingless bees that were added during the last year (Q 20);

#nests OUT—the number of nests of stingless bees that were sold, donated, or given away during the last year (Q 21).

To determine any relation between the NDR (independent variable) and the overall score (dependent variable), we made a linear correlation test to obtain the Pearson’s coefficient. To check the normality of the data (both overall score and NDR), a Kernel density estimation and a Shapiro–Wilk test were performed. A two-sample Wilcoxon rank sum test (Mann–Whitney) was used to test whether melipolicultors who had an NDR of less than 15% and an NDR equal or above 15% belonged to the same population or not.

### 2.4. Spider Web and Barometer Tools

For a general visualization of the status of meliponiculture, as an activity that must include minimum standards of compliance with GMPs in each group of questions, two tools were developed. The first one, the spider web tool, contrasts the status of each area: information sources, the application of basic biosecurity standards and the use of personal protective equipment, monitoring in health care, and conservation actions. For this purpose, we used the total score obtained per respondent and an average obtained per question group (see Table S2). The result (percentage) given for each group of questions indicates how closely the practices are aligned with what is expected according to scientifically based theoretical criteria. The closer the result is to 100%, the better the practices are considered, and the closer the result is to 0%, the more there is an opportunity for intervention and improvement in that area.

The second one, the barometer tool, ranks the overall status using the average of the above values. It means that from a global perspective, meliponiculture is evaluated and qualified. To determine the status, we divided the barometer bar into three zones, using quartiles (Q1 and Q3) of the overall score. Each zone has an action proposal, i.e., red zone: to write an action plan, implement it, and audit again within a month; orange zone: to take corrective actions and check their implementation; green zone: the management and practices are the best. 

## 3. Results

**Figure 1:**
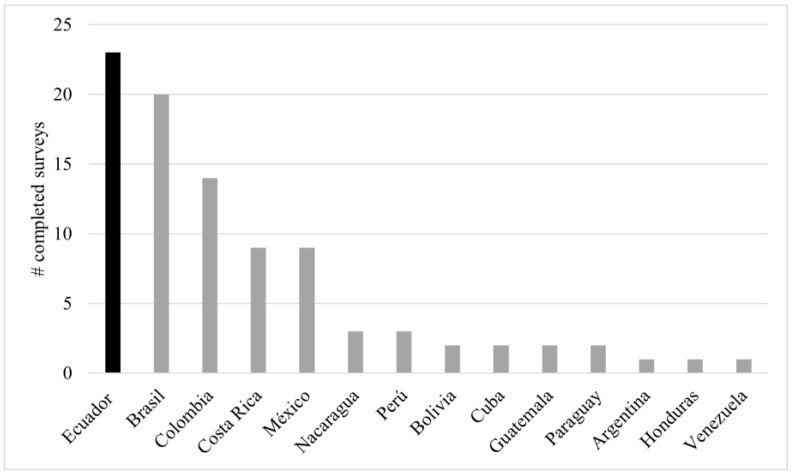
Survey participation by country. The bars represent the number of completed surveys (y-axis) per country (x-axis).

We collected a total of 94 surveys, of which only 92 were used because two were eliminated during data cleaning and validation. Surveys were collected from 14 Latin American countries (Figure 1). In terms of academic level, a university degree was obtained by the largest percentage of respondents (38%). The mean age of the respondents was 43 years. Experience as a stingless bee keeper ranged from 5 months to 52 years. An average of 48 nests per meliponicultor was calculated. The total number of nests among all respondents amounted to 4548 (by nest, the median = 17, min = 1, and max = 700). Most respondents spent part of their time (about 8 h per week) on the care and management of stingless bees. The individual product with the highest percentage of harvest was honey (16%), followed by a combined harvest that included honey, cerumen, pollen, and geopropolis (63%) (Table 1).

### 3.1. Environment and Conservation (GMP–CONSERV)

A total of 61% of stingless bee keepers consider that there are one or more sources of pollution around their nests. From the highest to lowest number of reports, there were plantations using agrochemical products for pest control, companies extracting oil and oil derivates (plastics), mining, city pollution (urban meliponiculture), and polluted rivers. In addition, 96% of respondents consider that climate change affects or will affect the life of bees. The same percentage of respondents take climate-friendly actions such as recycling, saving energy, not using agrochemicals for pest control, and planting more plants, and a small percentage of producers (n = 4/92, 4%) mention “agroecology” as a new climate-friendly practice.

The main reason for keeping stingless bees was the conservation of land (93%), pollinators, or biodiversity in general and the conservation of ancestral agricultural heritage in particular. Respondents (n = 21/92, representing 22%) purchased whole nests or brood disks to obtain more stingless bee nests. In general, those who buy nests try to get them from nearby areas (n = 10/21, representing 48%), same region (n = 6/21, representing 28%), or same country (n = 2/21, representing 9%), except in one case (n = 1/21, representing 4%) (international purchase). 

A total of 60% of stingless bee keepers feed their managed stingless bees with water, *Apis mellifera* honey, honey from other stingless bee species, commercial food, and processed substances such as sugar, flour, or lemon juice. They do it according to stingless bees’ necessity, i.e., breeding seasons, winter/non-flowering, new splits, weak nests/no reserves, and also for the maintenance and stimulation of nests.

### 3.2. Producer Training and Modern Techniques (GMP–TECHN)

To obtain their first nest, 76% of respondents practiced trapping in the wild. It is important to notice that some other meliponicultors (8%) obtained their first nest by rescuing stingless bee nests that were in significant danger. Respondents (37%) mentioned that they received expert support or some previous training for the transfer of natural nests to wooden boxes for technical nest management. However, a percentage of respondents (n = 12/92, representing 13%) keep nests in natural structures (i.e., hollowed tree trunks).

During nest division, stingless bee keepers confirmed that they ensure the following conditions: the existence of a viable virgin queen and old virgin, the health of and sufficient food for the old nest and the new nest, the seasonal flowering of plants (summer), positioning the new nest and scheduling the time of bees’ work that avoids damages or loss of workers, the existence of mature–viable brood discs, an abundant population, and a strong and disease-free nest of origin. Excluding urban meliponiculture, 92% of the producers maintain their nests in open spaces with plants. 

The organization of stingless bee nests (meliponaries) was attributed to being specific to the species managed, the size of the bees, their behavior, and the ease with which the nests can be harvested. The most reported conditions are described as follows: at least 1 m above the ground, one nest next to the other, minimum separation between nests of 0.40 to 3 m, nests stacked one on top of the other (condominium or tower blocks), and nests directly on the ground. This survey did not ask species-specific questions about nest organization in a meliponary; thus, the conditions detailed above are a general guide.

Among the places where respondents located their meliponaries were their own land (n = 61/92, representing 67%), common land (n = 21/92, representing 23%), association land (n = 4/92, representing 4%), natural tourist spaces (n = 4/92, representing 4%), and land belonging to academic institutions (n = 2/92, representing 2%). 

Academia is the main source of producer training or teaching (n = 57/92, representing 62%). Knowledge sharing among producers is strong (around 28%), with social networks being the main channel of information transfer, where experienced meliponicultors share their knowledge with those who are new to the activity.

### 3.3. Use of Personal Protective Equipment and Biosecurity Practices (GMP–BIOSEC)

One person manages the meliponary in 73% of the cases, while 27% of respondents stated that they do not carry out meliponiculture alone. The accompaniment for activities in the meliponary ranged from 2 to associations of 25 people (Ecuadorian example).

**Figure 2:**
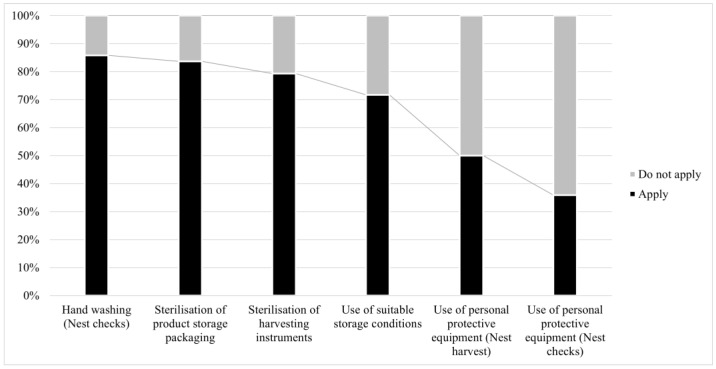
Application of basic biosecurity standards in stingless bee nests. Percentage of compliance (y-axis) with specific biosecurity standards in stingless bee nests (x-axis). Ordered from highest to lowest and differentiated by stage during nest management.

The application of biosecurity practices and the use of appropriate materials are summarized in Figure 2. It is important to mention that hand washing and the use of personal protective equipment (PPE) during regular nest checks had the same behavior in both management cases (one person or more than one person). The main PPE and instruments used for different activities at the surveyed meliponaries are summarized in Table 2. The use of a sterilized material for product storage (n = 75/92, representing 82%) as a biosafety measure was also emphasized in the survey. The main storage conditions for products were as follows: refrigeration (4 °C) (n = 34/75, representing 45%), protection from humidity (n = 25/75, representing 33%), protection from light (n = 12/75, representing 16%), and environmental temperature and freezing (−20 °C) (n = 4/75, representing 5%).

### 3.4. Health Care (GMP–HEALTH) 

**Figure 3:**
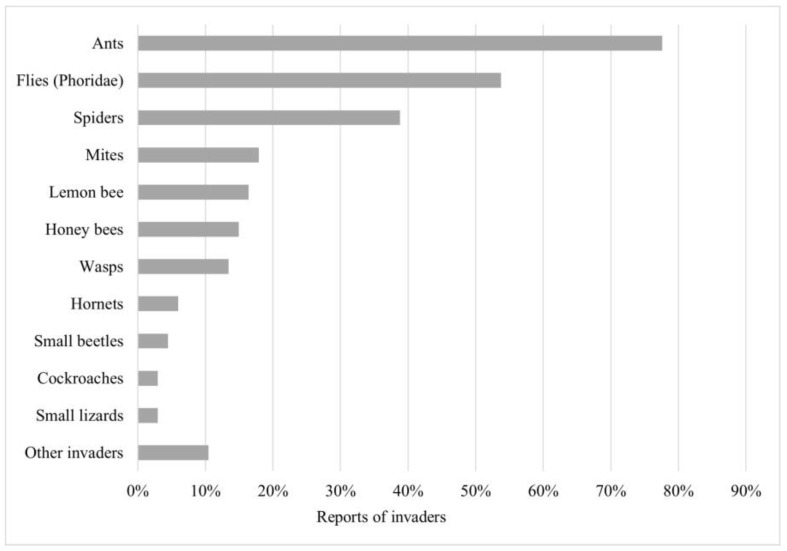
Presence of invaders in stingless bee nests. List (y-axis) and percentage of stingless bee nest invaders reported (x-axis). Sorted from highest to lowest number of reports. Other invaders include just one report of Euglossini and Bombini bees, crickets, mammals, blank soldier fly (*Hermetia illucens*), termites, and arapuá bee (*Trigona spinipes*).

**Figure 4:**
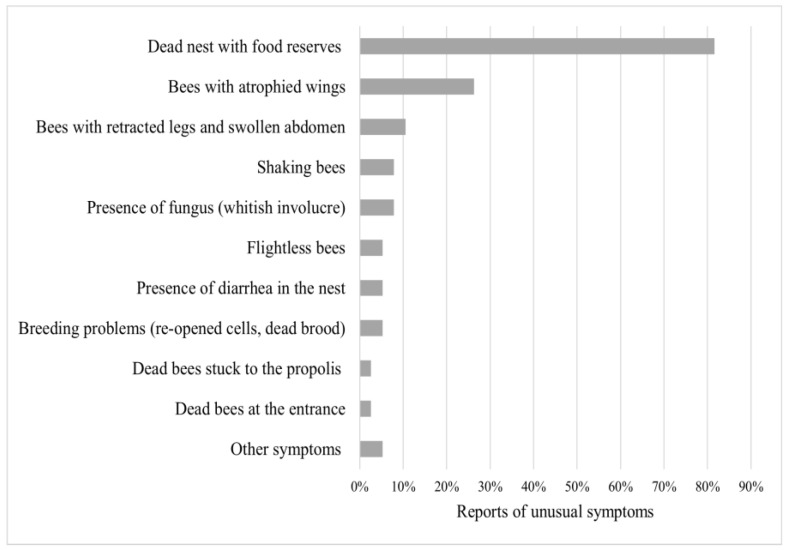
Presence of unusual symptoms in stingless bees. List (y-axis) and percentage of reported unusual clinical symptoms in stingless bees (x-axis). Sorted from most to least severe. Other symptoms include just one report of death by pesticides and invasion by the same species.

Meliponicultors (n = 62/92, representing 57%) kept a record of activities carried out in their meliponaries. In these records, they have been able to observe aspects such as insects/organisms invading stingless bee nests (73%) and unusual behavior (44%), detailed from the highest to lowest rates of sighting in Figure 3 and Figure 4.

The first place in terms of the most commonly reported invaders of stingless bee nests is occupied by ants, followed by Phoridae flies and spiders. The two best-known problem insects for meliponiculture are the phorid fly and the lemon bee (ranked fifth in this study as an invader).

Respondents (n = 28/92, representing 30%) know about nosemosis (no statistically significant effect on NDR, Mann–Whitney test, *p*-value = 0.262). More than half of the total respondents (n = 52/92, representing 57%) confirmed the existence of apiaries near their meliponaries (no statistically significant effect on NDR, Mann–Whitney test, *p*-value = 0.733). Knowledge of nosemosis was not associated with the existence of honey bees near stingless bee nests (no statistically significant correlation between the variables in question, Pearson product–moment correlation test, *p*-value = 0.219). 

Only one meliponicultor replied that he treated his bees with veterinary medicine and did not store this medicine after it was opened (this survey did not collect data regarding the specific type of medicine employed by stingless bee keepers for the treatment of their bees). Among the sources of reference to face and solve unusual health concerns in nests, the meliponicultors answered that 69% prefer to ask other stingless bee keepers, 13% consult an expert (veterinarian), another 13% prefer to experiment by themselves, 9% treat the bees by themselves since they have previous knowledge, and a small 1% go to academic bibliographic sources or theses.

### 3.5. Relationship between the NDR and the Application of Good Practices in Meliponiculture

**Figure 5:**
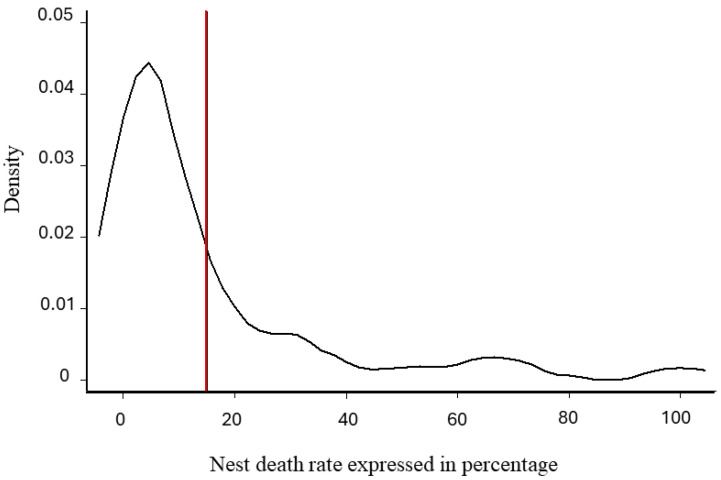
The kernel density estimate of the nest death rate. X-axis: probability density. Y-axis: nest death rate calculated and expressed as a percentage (scale between 0 and 100%). The red vertical line at 15% represents the observed cut-off point to separate the population into two parts.

Normality was verified for the overall score (dependent variable) (Shapiro–Wilk test, *p*-value =0.614 for theoretical best score, *p*-value =0.617 for best meliponicultor score) but not for the NDR (independent variable), giving us a cut-off point = 0.15 (i.e., 15%), which divides the population into two groups based on nest losses (Figure 5).

**Figure 6:**
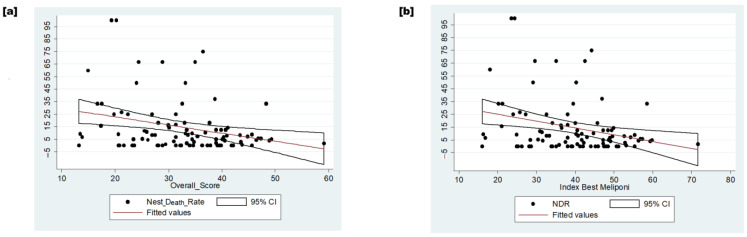
The relation between dependent and independent variables. (**a**) The inverse relation between the overall score and nest death rate. (**b**) The inverse relation between the index of the best meliponicultor and the nest death rate. Legend: NDR—nest death rate.

An inverse relationship was observed between compliance with GMPs and NDR (Figure 6). The linear correlation between variables explained 8% of the NDR concerning the overall score (*p*-value = 0.005).

**Figure 7:**
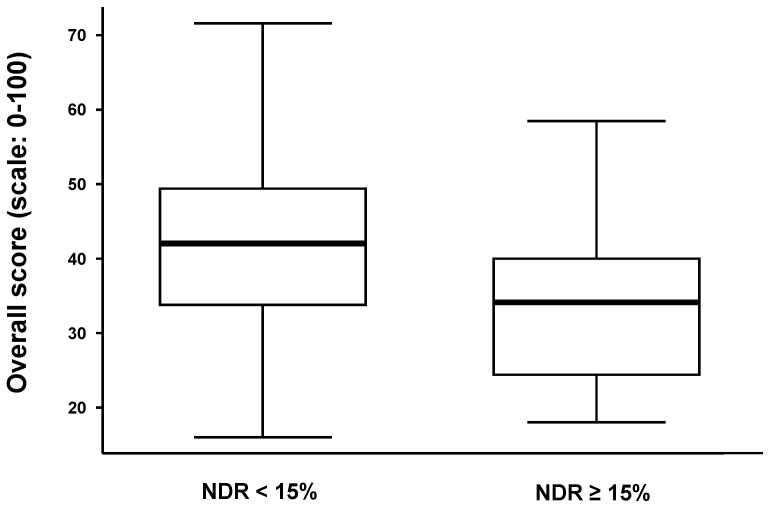
A boxplot of the overall score (y-axis) vs. the estimated nest death rate (x-axis). Population division is visualized considering the estimated mortality rate. NDR: nest death rate. Legend: The horizontal bold line in the rectangle represents the median of the overall score; the solid lines at the top and bottom of each rectangle represent, respectively, the first and third quartiles; adjacent lines to the whiskers represent the limits of the 95% confidence interval.

The overall scores are significantly different in the two sub-groups of meliponicultors depending on the NDR and considering the cut-off point of 15% (Mann–Whitney test, *p*-value = 0.001) (Figure 7). The last three calculations were verified by both methods using the best theoretical and best meliponicultor scores.

### 3.6. Spider Web and Barometer Tools

**Figure 8:**
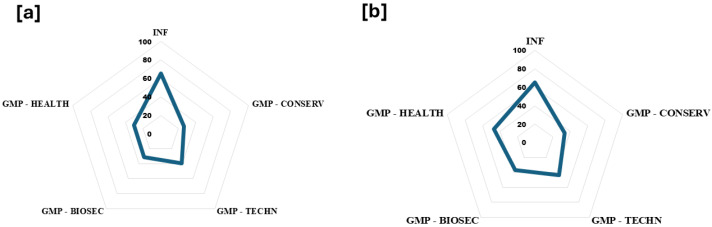
Spider tool. Overview of compliance in each analyzed area: social aspects, modern techniques, health, biosafety, and conservation. (**a**) The percentage of compliance based on the theoretical best score. (**b**) The percentage of compliance based on the score obtained by the best meliponicultor. INF: socio-demographic information. GMP: good management practices. CONSERV: environment and conservation. BIOSEC: biosecurity measures. TECHN: producer training and modern techniques.

The spider web tool showed a great socio-demographic status (65.4% of compliance). Items better aligned with scientific theoretical criteria, from the highest to lowest percentage of compliance were as follows: GMPs applied to training and modern techniques, GMPs in healthy controls, GMPs in biosecurity practices, and environmental protection actions (Figure 8a). However, when it is differentiated by the best meliponicultor score, GMP—HEALTH comes in second place, followed by GMP—TECHN, GMP—BIOSEC, and GMP- ENV PROTEC (Figure 8b).

**Figure 9:**
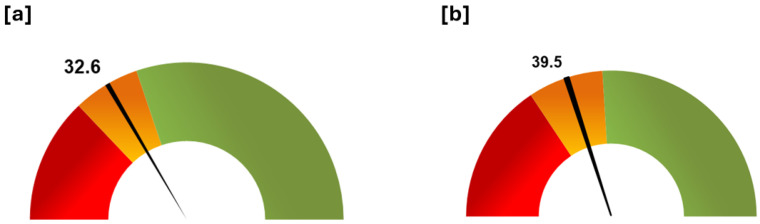
Barometer tool. Summary of the general status of the stingless bee keeper respondent population in terms of good management practice compliance. (**a**) Status based on the theoretical best score (Q1 = 25.8, Q3 = 39.6). (**b**) Status based on the score obtained by the best meliponicultor (Q1 = 31.3, Q3 = 48.03). Legend: The barometer was divided in three zones, using quartiles (Q1 and Q3) of the overall score. Each zone has an action proposal, i.e., red zone: to write an action plan, implement it, and audit again within a month; orange zone: to take corrective actions and check their implementation; green zone: the management and practices are the best.

The barometer tool gave a result of 32.6% for the theoretical best score (Figure 9a) and 39.5% for the best meliponicultor (Figure 9b), both right in the middle of the orange zone, which asks respondents to take corrective actions and check their implementation.

## 4. Discussion

This pilot study mainly reached a “sector” of the stingless bee keepers population with access to the internet, a cell phone, or a computer, as well as to studies, which is reflected in the highest percentages of respondents with university and high school education, which may be surprising given the rural reality of the world. In Ecuador, a 2019 study showed a shift in university enrollment among rural youth in a coastal province, largely due to the confidence parents now have in university education [37]. The rise in student demand for distance education has reached 10% per semester, an alternative modality to solve the problem of remote locations, through a system of grants for the implementation of technology at home.

It is important to note that the current statistics about education enrollment do not reflect the reality of the entire rural youth population of Ecuador, let alone Latin America. However, they do provide an approximation of meliponiculture and the potential loss of its ‘rurality’ in the context of a globalized world. This could potentially result in the loss of ancestral knowledge on meliponiculture, which has been practiced for a considerable length of time [38,39], more than 2000 years [11].

Furthermore, the utilization of technologies, such as these online surveys, facilitated the gathering of data and insights into the contemporary practices and management of meliponiculture. A significant approach was to gain an understanding of the processes involved in the care of stingless bee nests, which is predominantly a collective endeavor involving family members or associations. Thus, knowledge is still inherited, and teamwork [25] helps to reduce errors, since each person assumes a single task.

The survey also shows the participation of the academy with the provision of institutional lands as a strategy for mutual benefit between producers and research. This community work extends knowledge among stingless bee keepers [40]. The hybridization between traditional knowledge and modern stingless beekeeping improves local practices, thus increasing production. If this were the case, above all, it would reduce the chance of colony losses [41].

This study highlights the role of more experienced meliponicultors, since they become sources of new knowledge and promoters of stingless beekeeping. While these examples of collaborative behavior and knowledge transfer are commendable, there is a need to recognize the continued risk associated with the perpetuation of less ethical practices in this field, especially risks associated with the introduction of animal or plant species (nectiferous) that may facilitate the spread of diseases or new predators/competitors. This is exemplified by the case of African tulips [42].

The mean age of stingless bee keepers as well as the variability in years of experience in this study compares with another Ecuadorian study [43], with ages from 22 to 72 years old, and with the average age of Brazilian meliponicultors being 44.1 ± 2.14 for women and 43.4 ± 0.78 for men, including 5.9 ± 0.5 years of experience in stingless beekeeping [44].

As a field activity, stingless beekeeping is a side job in families that practice it, even though the marketing value of honey is around USD 133–200/Kg [45]. As it is a secondary activity, people invest 8 h per week on average. Taking time between revisions helps to keep nests free of pests. Even in critical periods, such as the time after the split, experts recommend checking the new nest every three days for three weeks, and then once a week [46], but above all, meliponicultors should not over-manipulate the brood comb [47].

### 4.1. Environment and Conservation (GMP–CONSERV)

Regarding stingless bee conservation aspects, a low percentage of respondents purchase nests from outlying areas from meliponaries. However, interregional and one international sale were reported in this survey, making it imperative to create awareness programs on the impact of colony displacement. The consequences of anthropogenic nest displacement have been widely reported [28,48,49].

Feeding stingless bees is appropriate at specific times, i.e., after honey harvest (low nutritional reserves) [50], during non-flowering seasons or harsh winters [51], to strengthen colonies after a split [52], and under pollination greenhouses [53], as well as the cases of urban meliponiculture found in this study. Feeding may include nectar (energy source) or pollen (protein source) replacement, such as the protein substitute in the diet of *Melipona flavolineata* that was tested and accepted under laboratory conditions [54]. 

It is our contention that the utilization of flour as a pollen substitute in stingless bees is a matter of concern. A study was conducted to evaluate the acceptance of four types of flours in a mixture of honey and water by honey bees. The results demonstrated that all mixtures were accepted, with soybean meal being the most accepted [55]. The quality of nutrition is associated with alterations in the gut microbiota of honey bees, which in turn impact their immune system and susceptibility to pathogens [56]. The impact of flour as a protein substitute in stingless bees remains largely unstudied.

### 4.2. Producer Training and Modern Techniques (GMP–TECHN)

Producers who followed training courses in meliponiculture were able to make nest divisions and provide adequate supplementary feeding according to the nests’ needs [52]. Good nest management depends mainly on the practice and continuity with which it is practiced and the support that can be provided by the academy [57] or field technicians.

A disadvantage of maintaining nests in their natural structures, i.e., tree logs, is difficulty during honey harvesting and the possibility of contamination, as it passes through waste areas [50]. In addition, shaking and turning the nest upside down to let the honey fall by gravity induces the loss of eggs that sink in the larval food, causing nest collapse [57]. Thus, the management suggestion is the use of technical boxes with vertical divisions and separate cavities for the brood chamber as well as for honey and pollen pots so that during the honey harvest, only the storage modules are removed and it would be possible to continue using the gravity honey harvesting technique.

In the case of Mexican “jobones”, whose structures are horizontal, single-story structures for brood chambers and food storage, the technique of gravity honey harvesting has been used since the pre-Hispanic Mayas [38] with no major reports of brood collapse. It is therefore possible to attribute this to the density of larval food and suggest that the bee larvae do not ‘drown’ but remain afloat for a certain time during the gyrus downwards from the nest for harvesting. This last topic merits further in-depth study, as well as the application of vacuum pumps or automated suction devices for honey extraction reported in this study.

The artificial division of colonies is recommended once a year [52]. Among the precautions to be taken during the division of nests are that the nest of origin must have abundant brood discs, a large population, and reserves of honey and pollen [46]. It should be performed at night or in an enclosed space with a mosquito mesh to avoid fly (Phoridae) infestation [58].

A 50/50 method for nest multiplication is being practiced [59]. Thanks to this study, it is possible to add the following suggestions: First, 4–6 brood disks should be transferred to the new nest. In species that build a queen cell, it is recommended that this queen cell should be included in one of the brood disks. It is preferred to feed the new nest 24 h after being transferred and to check it at least twice a week. It is not recommended to transfer pots of honey or pollen in poor conditions [60,61]. All these considerations contribute to making the propagation techniques sustainable and self-sufficient because they will always have new queens available [62].

Trap nests are considered a viable tool to study stingless bee colonies for meliponicultors, researchers, and conservationists [54]. Traps are used to identify species and differentiate their distribution in primary and degraded forests [63]. The use of traps should not be for the over-exploitation of natural resources, as this may generate a disturbance in the ecological balance [64].

The primary motivation for engaging in meliponiculture was conservation, while the primary source of meliponicultors’ initial nests was through trapping. This does not necessarily indicate a contradiction but rather a potential deficiency in understanding the true nature of conservation. Trapping may potentially contribute to the unnecessary extraction of stingless bee nests from the wild. The removal of nests from their natural habitat should only occur when stingless bees are at risk, e.g., due to deforestation. 

### 4.3. Use of Personal Protective Equipment and Biosecurity Practices (GMP–BIOSEC)

The implementation of biosecurity measures on a farm prevents the introduction and spread of infectious agents and diseases [65]. For example, the use of personal protection equipment and hygiene were considered protective factors against colony loss in Belgian beekeeping [66]. The use of personal protective equipment as well as sterilized instruments are keys to improving nest management because stingless bee keepers can focus their attention on an activity free of bites or any discomfort that these species can cause [67].

The maintenance of colony hygiene is directly correlated with the safeguarding of bee health and the protection of bee products. Disinfection represents a hygienic measure that is designed to prevent and eliminate agents that are capable of causing infectious diseases in bees. Furthermore, it serves to avoid the contamination of honey and other bee products with harmful microorganisms [68]. Given the toxicity and other negative effects of chemical disinfectants, it is recommended that physical methods of disinfection be employed wherever feasible. 

In regard to physical methods of disinfection, the following is recommended for implementation in the field: boiling the instruments in water at normal (atmospheric) pressure for a period of 30 min. It is recommended that instruments be washed with hot water at a temperature of 90 °C or use hot air (110 °C and 150 °C) [69].

Stingless bee honey is characterized by having high moisture in comparison with *A. mellifera* honey, causing a natural fermentation process [70]. This fermentation process made by symbiont microorganisms contributes to the preservation of honey and the transformation of pollen into bee bread [71]. The findings of this study allow us to propose storage conditions for honey: refrigeration (4 °C) and containers that protect from humidity and light.

### 4.4. Health Care (GMP–HEALTH)

Local experts in Mexico reported attacks on stingless bee nets by different predators [72]. Indeed, the list of predators includes skunks (*Mephitis* sp), *Canis latrans*, *Dasypus novemcinctus*, ants, wasp rams, kleptobiotic stingless bees (*Lestrimelitta chamelensis*), and *A. mellifera*. Some of those predators were reported in this study in Brazil, Costa Rica, Colombia, Ecuador, and Perú.

Both Phoridae flies (*Pseudohypocera kerteszi*) and *Lestrimelitta* sp. can cause the complete loss of stingless bee nests, but the Phoridae fly is considered the most representative risk as far as stingless bee plagues are concerned. At least, *Lestrimelitta* sp is considered a biological population controller of stingless bees. Therefore, the recommendation that is under the control of the stingless bee keepers is the maintenance of hygiene in the nests, especially at the beginning of a transfer from a natural nest to a technical one.

For Phoridae flies, a useful recommendation is to collect all honey and pollen from the nest pots to prevent fly eggs from hatching and to constantly check these three areas of the nest which are the favorite places to start an invasion, and the use of white or red vinegar traps inside the nests [73].

Unusual signs in stingless bees such as extended proboscis, expanded or unhooked wings, wrinkled bodies, and defecation on cage covers are visible signs of poisoning with some agrochemicals (e.g.,): fipronil, cypermethrin, dimethoate, imidacloprid, and indoxacarb [74]. Crippled wings and a contracted abdomen are visible indicators of a possible infection with deformed wing virus (DWV), Israeli acute paralysis virus, and Kashmir bee virus (KBV) [75]. Trembling movements in bees and the inability to fly are reported as signs of acute bee paralysis virus (ABPV) infestation [76]. These unusual behaviors raise alarm bells regarding the health of stingless bees since their signs are similar to those described in *A. mellifera*. However, there are no reports in native bees, except for *Vairimorpha ceranae (Nosema ceranae)* [77]. 

In this study, the reported proximity of *Apis mellifera* to meliponid sets may present a risk to the health of stingless bees, given the potential for their interaction in the same floral resource during foraging [14]. It has been demonstrated that honeybee pollen loads frequently contain pathogenic protozoa and microsporidia [78]. The utilization of this pollen as a food source for stingless bee nests suggests a heightened probability of the transmission of infectious agents. Nevertheless, research has demonstrated that propolis derived from stingless bees can effectively mitigate the progression of *Nosema* infections in honey bees [79]. It is possible that propolis, a resinous substance used by stingless bees in the construction of their nests, may offer protection against *Nosema* infection.

It is therefore recommended that the use of honey bee products in stingless bee nests be avoided. In cases where the use of such products is unavoidable and within the reach of stingless bee keepers, it is advised that they verify that the products do not contain any agents or substances that could prove harmful to the stingless bees.

The natural ecology of stingless bees includes natural biological controllers such as lemon bees and phorids [80], as well as their natural competitive relationships, such as fights with solitary bees for resources [81]. These examples also cause morphological damage and even death to stingless bees. It is recommended to examine this symptomatology in depth and make accurate diagnoses of possible viruses or bacteria that are pathogenic to native bees.

Registering activities such as unusual behaviors, invasions, death, and other aspects in the meliponary [25] can be used as a basis for creating or providing records that can be submitted to or socialized with legal entities for regularization and health surveillance purposes.

### 4.5. Developed Tools

The annual calculation of the death rate under technical management conditions and without considering the difference in calculation between forage and non-forage stingless bees compares with the natural nest death rate reported at 13% for stingless bees [82] and 10% for honey bees [83]. Therefore, this value of the death rate in stingless bees should be considered an acceptable level of colony loss rates under domestic management. It was also verified that the better the compliance with good management practices, the lower the loss or mortality (inverse relationship).

The three main groups of causes associated with an increase in nest loss, namely GMP-CONSERV, can be attributed to two key factors: the high prevalence of polluting sources in close proximity to the meliponaries and the growing consensus regarding the adverse impact of climate change. Additionally, the GMP-BIOSEC group is included due to the dearth of adherence to fundamental biosecurity standards during nest inspection and product harvesting. This is a significant concern for the preservation of nest health and the quality of the products obtained. Finally, the GMP-HEALTH group is of note for the high number of reports of nest-invading insects causing nest collapse, as well as the observation of unusual behaviors in bees. These observations are comparable to those made in honey bees, but it is unclear whether the same causal and effect relationships can be applied to stingless bees. 

The spider web and barometer tools are pedagogic instruments to interact with meliponicultors and identify margins of improvement. The interpretation of the spider tool means that the sources of information, experience, and management practices of meliponicultors are alienated to extend stingless bees’ life, as well as environmental protection, according to scientific theoretical criteria. At the same time, the barometer tool confirms the widely discussed need for the implementation of good management practices.

The benchmarking made for score assignment showed that meliponiculture should have its guidelines, and even within meliponiculture, management should be separated according to the stingless bee species being managed, according to the region where the activity is developed, and according to the scientific information that each country generates.

The limitations of the present pilot study can be attributed to the continuous growth of meliponiculture and therefore research, since we only have three examples of developing tools for the evaluation of stingless beekeeping, in Mexico, Brazil, and Costa Rica. People dedicated to this activity are located mainly in rural zones, and the lack of access to internet sources (the main medium of dispersion of this pilot survey), is a limitation. The reliability that researchers can create with producers must be considered. 

Despite evidence of the positive influence of the training and education of stingless bee keepers, more programs of this kind should be created or research results should be disseminated in the language of stingless bee keepers and on freely accessible platforms, as a large percentage base their management practices on the advice of others meliponicultors. Improved management and risk control in meliponiculture should be addressed using this economic activity as a tool inside agroecological systems. A loss/death rate calculation will improve long-term nest management conditions. Finally, we recommend the application and socialization of spider and barometer tools with meliponicultors in the field through an app.

## 5. Conclusions

Stingless beekeeping in Latin America, especially in Ecuador, is growing rapidly. Fortunately, guidelines related to biosecurity show acceptable nest management. However, some items need to be addressed to ensure better health: global compliance with biosecurity measures, actions for the care of the environment in which stingless bees live, the quality and efficiency of technology in the handling and management of nests, and the diagnosis/monitoring of the health status of stingless bees.

Hand washing and sterilization are applied during management and constitute a very good basis for turning meliponiculture into a sustainable practice.

Risk factors for the conservation of stingless bees include the effect of the introduction of species such as the European honey bee as a potential disease disperser, the use of agrochemicals, the pollution that bees face, and the effect of anthropogenic activities such as colony movement that are not aligned with good management practices.

Honey, as the main product harvested, must have an adequately good management procedure from harvesting to storage, due to its unique physical and chemical characteristics. However, it can become complex as the number of nests increases.

The nest death rate calculated here does not exceed the naturally calculated rate by far. It is a good indicator that the human practice is performed in a good way. However, the application of practices that were found to be missing in this study could reduce this percentage to a more acceptable number.

Graphic tools such as the spider and the barometer are instruments for the empowerment of each meliponicultor, as they help in the field and instantly help detect shortcomings to be corrected after entering some parameters.

## Figures and Tables

**Table 1:** Summary of the main socio-demographic variables.

Variable	Range		Percentage
Age (years)	Young	≤28	25
	Adult	>28 and ≤60	63
	Old adult	>60	12
Stingless beekeeping experience (years)	Beginner	≤5	54
	Upper beginner	>5 and ≤10	18
	Intermediate expert	>10 and ≤20	16
	Expert	>20	11
Full academic level	Elementary		1
	High School		29
	Technology		12
	University		38
	Post grade		20
Spending time	Full Time (≥8 h/day)		9
	Part-time (<8 h/day)		23
	Hobby (~8 h/week)		68
Amount of nests (quantity)	≤10		34
	>10 and ≤50		47
	>50 and ≤100		8
	>100		12
Main product harvested from nests	Honey		16
	Geopropolis		4
	Cerumen		3
	Honey, cerumen, geopropolis, pollen		63
	Other reason for nest keeping *		13

* Among other reasons for keeping nests of stingless bees were (i) nest multiplication for sale, (ii) stingless bee conservation, and (iii) protection.

**Table 2:** Summary of the main biosecurity measures complied with in the key stages of meliponiculture (regular check, harvesting, product storage).

**Item**	**Activity in the Nest Set (Meliponary)**	
	Regularly Check (n = 33)	Harvesting (n = 45)
(a) Personal Protective Equipment		
Head coverings	30	18
Sterile gloves	15	28
Face mask	9	31
Clean boots	9	
Clothing cover	10	
Protective glasses	5	
Tent for creating a sterile environment		5
	Harvesting (n = 71)	Product storage (n = 75)
(b) Instruments		
Food-grade containers	45	
Spoons or paddles	31	
Syringes	51	
Filters	41	
Palette, knife, scrapers	7	
Vacuum pumps	3	
Glass bottles with lids		66
Plastic bottles with lids		24
Plastic bags with hermetic seals		8
Glass bottles with gas release		1

## Data Availability

The data that support the findings of this study are available from the corresponding authors upon reasonable request.

## Supplementary Materials

The following supporting information can be downloaded at: https://www.mdpi.com/article/10.3390/insects15090715/s1, Table S1: Dispersed online questionnaire ‘Good management practices for stingless bees’. Table S2: Example of calculation of the score by group of questions and the overall score.
