# Supplementary material for: Development of Tools to Understand the Relationship between Good Management Practices and Nest Losses in Meliponiculture: A Pilot Study in Latin American Countries
Source: Insects. 2024 Sep 19;15(9):715. doi: 10.3390/insects15090715 (PMC11432252; doi:10.3390/insects15090715)
Supplement: Supplementary file 1 [file insects-15-00715-s001.zip › insects-3174414-supplementary.pdf]

Table S1. Dispersed online questionnaire 'Good management practices for stingless bees'. The original language of the survey was Spanish and the only translation was into Portuguese.

GOOD MANAGEMENT PRACTICES FOR STINGLESS BEES
The aim of this survey is to find out what practices you have or have had during your stingless bee nest management, habits from obtaining a new nest to harvesting products from the nest. This survey will not collect sensitive personal information. The data collected here will be used for scientific purposes, in the framework of the research project developed by Sofia Ocaña, Ecuadorian by birth and PhD student at the University of Liège - Belgium. Please read and answer each question carefully. The approximate time it will take you to complete the survey is 7 minutes.
**GENERAL RESPONDENT INFORMATION**
1. Date
2. Country
3. City
4. Respondent's date of birth
5. Maximum level of education completed by the respondent
Primary<br>Secondary<br>Technology<br>University<br>Postgraduate degree<br>None
6. What is your main interest in keeping stingless bee nests?
Own consumption of the nest products<br>Sale of nest products<br>Conservation of biodiversity<br>Conservation of pollinator lands and flora<br>Encouraging tourism/educational tourism<br>Conservation of the culture<br>Other, please specify below
7. As a honey beekeeper, where do you get information on stingless bee guidelines or updates?
Workshops/courses of the Ministry of Environment of the country<br>Workshops/courses of the Provincial/Municipal Environment Directorate of the city<br>Beekeeping technicians, stingless bee experts, or veterinarians<br>TV, radio, or national newspaper<br>Beekeeping and/or meliponiculture congresses<br>Through information exchange with other beekeepers, meliponicultors, or community<br>None of the above<br>Other, please specify below
8. Please indicate your experience (years) as a stingless beekeeper.
9. How many hours a day do you spend caring for, cleaning or checking the nest(s) you own?
Full-day major o equal than 8h per day<br>Part day less than 8h per day<br>Per trade or hobby around 8h per week
**GENERAL INFORMATION ON NEST MANAGEMENT AND PRODUCTS**
10. Are you the only person who manages the meliponary that you own?
YES

NO
10.1. Please indicate the total number of persons involved in the management of the meliponary.
11. Do you use protective equipment during the regular checking of your nests?
YES<br>NO
11.1. Do you know if the other persons involved in nest care use protective equipment during nest care and checking?
YES<br>NO<br>DOES NOT KNOW
11.2. Please select the type of protective material that you or your support staff use during nest care and checking
Sterile gloves<br>Goggles<br>Mask<br>Clothing protector/coverall<br>Clean boots.<br>Head covering or veil.<br>Other, please specify below
12. You wash your hands before checking the nests
YES<br>NO
12.1. Do you instruct your support team to wash their hands before checking nests?
YES<br>NO
13. Which of the following products do you collect from your meliponary?
Honey<br>Pollen<br>Propolis<br>Wax<br>Cerumen<br>None of the above<br>Other, please specify below
14. Do you use personal protective equipment during the harvesting of products?<br>YES<br>NO
14.1. Select the type of personal protection you or your support staff use during the harvesting of produce
Sterile clean gloves<br>Face shields/Mask<br>Medical caps<br>Tent creating an isolated environment for harvesting<br>Other, please specify below
15. Do you use pre-sterilized instruments during the harvesting of produce?
YES<br>NO
15.1. Select the type of instrument you or your support staff use during product harvesting

Syringes<br>Pipettes<br>Spoons<br>Wooden pallets<br>Sieve/sifter/strainer<br>Stainless steel plates<br>Food grade trays<br>Other, please specify below
16. In relation to harvested produce do you use pre-sterilised instruments for storage?<br>YES<br>NO
16.1. Please select the materials you or your support staff use to store the products /
Glass jars with lids<br>Plastic jars with lids<br>Tightly closing pouches<br>Pouches<br>Other, please specify below
16.2. Indicate the conditions that you or your support staff maintain during the time of storage of the products
Refrigeration (4 °C)<br>Freezing (-20 °C)<br>Protected from light<br>Protected from humidity.<br>No conditions were used during the storage of products<br>Other, please specify below
17. Do you keep a record of the activities you carry out in the meliponary, or of the treatments you apply to the nests (in case of sickness)?
YES<br>NO
**NEST ORIGIN AND NEST DIVISION, MELIPONARY INFRASTRUCTURE**
18. How did you get your first stingless bee nest?
Buy brood discs<br>Catch a nest that was found in the wild<br>Bought complete nests<br>Other, please specify below
18.1. Another source of your first stingless bee nest, please specify which one
18.2. If you have obtained at least one nest from the wild, please answer
Keep the nest in the container in which you found it<br>Have you changed/moved the nest to a technified wooden box<br>Have you changed/moved the nest to clay pots
18.3. If you have made changes/moves from a nest to a technified wooden box or clay pot, please answer
You received help from an expert<br>You already knew how to do it<br>You decided to experiment/try it yourself<br>You have not made any changes/moves to nests<br>Other, please specify below

19. How do you get more stingless bee nests?
You divide nests you already have<br>You buy new nests<br>You get new nests from the field / nature<br>You set traps to catch stingless bee colonies in the field/nature<br>Other, please specify below
19.2. If you buy new nests, please specify the place from where you get them / place of origin.
19.3. If you split your nests, which of the following aspects do you take into account or consider during this activity
The brood discs of the new nest have at least one cell with a virgin queen<br>The nest of origin is left with a functional queen available<br>The nest of origin is left with sufficient honey and pollen<br>The new nest has enough pollen<br>The nest of origin is left with at least 3 brood discs after the splitting<br>The nest of origin is not left with any health and/or production problems<br>None of the above<br>You do not divide the nests<br>Other aspects during the division of nests, please specify below
20. How many nests in total have you added during the last year to your meliponary?
21. If your meliponary has the capacity to produce and sell stingless bee nests, please indicate how many nests in total you have sold/donated/given away during the last year.
22. Where have you located your meliponary
Backyard of your home<br>In your house<br>On the land/land of your property<br>On the land of a neighbour/friend/acquaintance<br>Other, please specify below
23. How do you organise/arrange / distribute the stingless bee nests?
Stacked one on top of the other<br>Next to each other<br>At least 1 metre apart<br>More than 1 metre apart<br>At least 1 meter above the ground<br>On the ground<br>Other, please specify below
24. Do you try to keep the stingless bee nests close to flowering plants?
YES<br>NO
25. Do you feed the stingless bee nests?
YES<br>NO
25.1. Do you feed the stingless bee nests with any of the following substances?
Water<br>Water with sugar<br>Bee bread and water<br>Commercial bee feeds<br>Pollen

Pollen and water<br>Honey from Honey Bees<br>Honey from stingless bees<br>Other, please specify below
25.2. Please describe the circumstances under which you feed the nests
The non-flowering time of the plants<br>When you have a new nest<br>Rainy season<br>Winter<br>Summer<br>Other, please specify below
25.3. Indicate the time during which you feed the stingless bee nests
Once a day<br>Twice a day<br>More than twice a day<br>Once a week<br>Twice a week<br>More than twice a week<br>Other please specify below
26. Indicate the number of stingless nests you currently own and manage
**HEALTH AND ENVIRONMENTAL ASPECTS OF NESTS**
27. Please indicate how many nests in total you had until 2021
28. Please indicate how many nests were lost/died during the last year
29. In your meliponary, have you ever observed insects or organisms other than stingless bees invading your nests?
YES<br>NO
29.1. Please select which insects you have observed invading your nests
Ant nests<br>Flies<br>Spiders<br>Mites<br>Fleas<br>Wasps<br>Avismonia<br>Melliferous bees<br>Other, please specify below
30. Please answer if you have observed the following "symptoms" in your nests
Bees with atrophied and/or deformed wings<br>Bees with tremors<br>Bees not flying or walking on the grass/grass<br>Dead bees with swollen abdomens and retracted legs under the thorax<br>Presence of diarrhea on the walls/floor of the nest<br>Nests lost/died but with honey reserves<br>Not observed any "symptoms"<br>Other, please specify below
31. Do you use any medication to treat any of the known conditions of stingless bees?

YES
NO
DON'T KNOW - DON'T REMEMBER

31.1. If used, do you keep medicines after they have been opened?

YES
NO

31.2. Select the storage conditions of the medicines you procure

Refrigerator (4 °C)
Freezer (-20 °C)
Vacuum
Protected from light
For other conditions, please specify them below

32. When you have treated your bees, have you done so under any of the following conditions

Consult a veterinary expert
You prefer to medicate your bees because you have prior knowledge
You consult other beekeepers
You prefer to experiment
For other conditions, please specify them below

33. Do you know about nosemosis?

YES
NO

34. Do you know about the existence of apiaries (honey bees) near your stingless bee nests?

YES
NO

35. Are you aware of the existence of the following sources of pollution near your nests?

Oil companies
Gas stations
Plasticizing companies
Plants using pesticides
Mining companies
Motorways
Hydroelectric power plants
There are no sources of pollution near your nests
Other sources, please specify below

36. Do you consider that climate change has affected or will affect the lives of stingless bees?

YES
NO

36.1. Please indicate if you have taken any environmental action

Recycling of rubbish
No use of pesticides (insecticides, herbicides, fungicides, etc.)
Composting of organic waste
Planted trees/plants
Reducing the use of plastics
Saving of lighting
Water saving
For other actions, please specify below

Thank you very much for your time and for answering the survey, if you have any questions, doubts, or suggestions please contact Sofía Ocaña by email at jocana@doct.uliege.be.

OK

Table S2. Example of calculation of the score by group of questions and the overall score.

Item		Total questions by item	# of questions used for the calculation	Max possible score by group question	Score obtained by a meliponicultor (example)	Score obtained by group question	Overall score obtained by meliponicultor
Demographical information	INF	8	2	7	5	= (5/7)x100	=[(5+100+90+122+39) /(7+124+142+167+51)]x100
Good Management Practices - Environment/ conservation aspects	GMP - ENV PROTEC	11	9	124	100	=(100/124)x100	
Good Management Practices - source of information and consulting	GMP - TECHN	7	6	142	90	=(90/142)x100	
Good Management Practices - personal protection equipment and biosecurity measures	GMP - BIOSEC	14	12	167	122	=(122/167)x100	
Good Management Practices - medical treatments, signals	GMP - HEALTH	10	9	51	39	=(39/51)x100	
